# Supplementary material for: Assessing the introduction risk of vector-borne animal diseases for the Netherlands using MINTRISK: A Model for INTegrated RISK assessment
Source: PLoS One. 2021 Nov 2;16(11):e0259466. doi: 10.1371/journal.pone.0259466 (PMC8562800; doi:10.1371/journal.pone.0259466)
Supplement: S1 Appendix — (DOCX) [file pone.0259466.s001.docx]

**S1 Appendix: Overview of input parameters in MINTRISK.**

| **Question number^a^** | **MINTRISK question** | **Input parameter for calculations** | **Answer type** | **Answer categories^b^** | | | | | **Log-transformation^c^** |
| --- | --- | --- | --- | --- | --- | --- | --- | --- | --- |
|  |  |  |  | Very low | Low | Moderate | High | Very high |  |
|  | Entry |  |  |  |  |  |  |  |  |
| **18** | Do epidemics of the disease occur somewhere in the risk region addressed? | *--* | Yes/No^d^ |  |  |  |  |  | No transformation |
| **19** | What is relative size of the epidemic area related to the full risk region?^f^ | *Area* | Quantitative ranges | < 0.01 | 0.01-0.03 | 0.03-0.1 | 0.1-0.3 | > 0.3 | ${10}^{\left( IV-1 \right)\times2.5}$ |
| **22** | What is the duration of the period (in years) between introduction of the infection into the region and notification of the infection (i.e. what is the length of the high risk period)?^f^ | *HRP_RR_* | Quantitative ranges | < 0.1 | 0.1-0.3 | 0.3 – 1 | 1 – 3 | > 3 | ${10}^{\left( IV-0.6 \right)\times2.5}$ |
| **23** | Are humans considered dead-end hosts? | *DEH* | Yes/No |  |  |  |  |  | No transformation |
| **24** | What is the frequency (per year) with which epidemics occur in the risk region?^f^ | *F_epi_* | Quantitative ranges | < 0.1 | 0.1 – 0.3 | 0.3 – 1 | 1 – 3 | > 3 | ${10}^{\left( IV-0.6 \right)\times2.5}$ |
| **25** | How high is the prevalence of the infection in host animals in the risk region at the end of the high risk period?^f^ | *Prev_epi_host_* | Quantitative ranges | < 10^-4^ | 10^-4^ – 10^-3^ | 10^-3^ – 0.01 | 0.01 – 0.1 | > 0.1 | ${10}^{\left( IV-1 \right)\times5}$ |
| **26** | How high is the prevalence of the infection in vectors in the risk region at the end of the high risk period?^f^ | *Prev_epi_vector_* | Quantitative ranges | <10^-5^ | 10^-5^ – 10^-4^ | 10^-4^ - 10^-3^ | 10^-3^ – 0.01 | > 0.01 | ${10}^{\left( IV-1.2 \right)\times5}$ |
| **27** | How high is the prevalence of the infection in humans in the risk region at the end of the high risk period?^fg^ | *Prev_epi_human_* | Quantitative ranges | < 10^-4^ | 10^-4^ – 10^-3^ | 10^-3^ – 0.01 | 0.01 – 0.1 | > 0.1 | ${10}^{\left( IV-1 \right)\times5}$ |

| **Question number^a^** | **MINTRISK question** | **Input parameter for calculations** | **Answer type** | **Answer categories^b^** | | | | | **Log-transformation^c^** |
| --- | --- | --- | --- | --- | --- | --- | --- | --- | --- |
|  |  |  |  | Very low | Low | Moderate | High | Very high |  |
| **28** | Is disease endemic somewhere in the risk region addressed? | *--* | Yes/No^e^ |  |  |  |  |  | No transformation |
| **31** | Are humans considered dead-end hosts? | *DEH* | Yes/No |  |  |  |  |  | No transformation |
| **32** | How high is the prevalence of the infection in host animals in the risk region?^f^ | *P_end_host_* | Quantitative ranges | < 10^-4^ | 10^-4^ – 10^-3^ | 10^-3^ – 0.01 | 0.01 – 0.1 | > 0.1 | ${10}^{\left( IV-1 \right)\times5}$ |
| **33** | How high is the prevalence of the infection in vectors in the risk region?^f^ | *P_end_vector_* | Quantitative ranges | <10^-5^ | 10^-5^ – 10^-4^ | 10^-4^ - 10^-3^ | 10^-3^ – 0.01 | > 0.01 | ${10}^{\left( IV-1.2 \right)\times5}$ |
| **34** | How high is the prevalence of the infection in humans in the risk region?^fg^ | *P_end_human_* | Quantitative ranges | < 10^-4^ | 10^-4^ – 10^-3^ | 10^-3^ – 0.01 | 0.01 – 0.1 | > 0.1 | ${10}^{\left( IV-1 \right)\times5}$ |
| **35** | What is the annual volume of animals / vectors / commodities / humans moved along the pathway from the risk region to the area at risk?^f^ | *V* | Quantitative ranges | < 100 | 100 - 10^3^ | 10^3^ - 10^4^ | 10^4^ - 10^5^ | > 10^5^ | ${10}^{\left( IV+0.2 \right)\times5}$ |
| **39** | How likely is it that viable pathogen is still present in the animal, vector, commodity or human upon arrival in the area at risk?^fh^ | *P_surv_transport_* | Quantitative ranges | < 10^-3^ | 10^-3^ – 0.01 | 0.01 – 0.1 | 0.1 – 0.95 | > 0.95 | If Answer=very high 1; Else  ${10}^{\left( IV-0.8 \right)\times5}$ |
| **41** | How likely is persistence / survival of infection in the animal, vector, commodity or human despite control / preventive measures during or after transport?^fh^ | *P_surv_PM_* | Quantitative ranges | < 10^-3^ | 10^-3^ – 0.01 | 0.01 – 0.1 | 0.1 – 0.95 | > 0.95 | If Answer=very high 1; Else  ${10}^{\left( IV-0.8 \right)\times5}$ |

| **Question number^a^** | **MINTRISK question** | **Input parameter for calculations** | **Answer type** | **Answer categories^b^** | | | | | **Log-transformation^c^** |
| --- | --- | --- | --- | --- | --- | --- | --- | --- | --- |
|  |  |  |  | Very low | Low | Moderate | High | Very high |  |
|  | Transmission |  |  |  |  |  |  |  |  |
| **45** | What is the distribution of the vector in the area at risk? | *D_vector_* | Patchy/Homogeneous/Unknown |  |  |  |  |  | No transformation |
| **46** | What is the estimated value of the basic reproduction ratio? | *R* | Quantitative ranges | < 0.3 | 0.3 – 1 | 1 – 3 | 3 – 10 | > 10 | ${10}^{\left( IV-0.4 \right)\times2.5}$ |
| **48** | Which fraction of the host population is susceptible to the infection (i.e. not protected from infection by routine vaccination or previous exposure)?^h^ | *F_susc_host_* | Quantitative ranges | < 0.03 | 0.03 – 0.1 | 0.1 – 0.3 | 0.3 – 0.95 | > 0.95 | If Answer=very high 1; Else ${10}^{\left( IV-0.8 \right)\times2.5}$ |
|  | Establishment |  |  |  |  |  |  |  |  |
| **50** | What is the probability of infecting a first local (indigenous) vector or host given the pathway of entry, and the expected region and time of entry? (first transmission step)^f^ | *P_inf_1_* | Quantitative ranges | < 10^-4^ | 10^-4^ – 10^-3^ | 10^-3^ – 0.01 | 0.01 – 0.1 | > 0.1 | ${10}^{\left( IV-1 \right)\times5}$ |
| **51** | What is the probability of infecting a first local vector (given first infection of an indigenous host) or host (given first infection of an indigenous vector)? (second transmission step)^fh^ | *P_inf_2_* | Quantitative ranges | < 10^-3^ | 10^-3^ – 0.01 | 0.01 – 0.1 | 0.1 – 0.95 | > 0.95 | If Answer=very high 1; Else ${10}^{\left( IV-0.8 \right)\times5}$ |
|  | Spread |  |  |  |  |  |  |  |  |
| **52** | What is the size of the (host) population at risk in the area at risk? | *PopS* | Quantitative ranges | < 10^4^ | 10^4^ – 10^5^ | 10^5^ – 10^6^ | 10^6^ – 10^7^ | > 10^7^ | ${10}^{\left( IV+0.6 \right)\times5}$ |
| **53** | What is the expected number of infection generations per vector season? | *IG_Season_* | Fixed number |  |  |  |  |  | No transformation |
| **54** | What is the overlap between (high) vector abundance and host density in the area at risk? | *Overlap* | Quantitative ranges | < 0.01 | 0.01 – 0.03 | 0.03 – 0.1 | 0.1 – 0.3 | > 0.3 | ${10}^{\left( IV-1 \right)\times2.5}$ |

| **Question number^a^** | **MINTRISK question** | **Input parameter for calculations** | **Answer type** | **Answer categories^b^** | | | | | **Log-transformation^c^** |
| --- | --- | --- | --- | --- | --- | --- | --- | --- | --- |
|  |  |  |  | Very low | Low | Moderate | High | Very high |  |
| **55** | To what extent does the presence of non-susceptible hosts in the area at risk result in a dilution effect? | *Dilution* | Quantitative ranges | < 0.01 | 0.01 – 0.03 | 0.03 – 0.1 | 0.1 – 0.3 | > 0.3 | ${10}^{\left( IV-1 \right)\times2.5}$ |
| **56** | To what extent is local spread in the area at risk inhibited by spatial effects? | *Local* | Qualitative ranges | minimal | little | moderately | largely | totally | No transformation |
| **57** | What is the expected length of the vector season (expressed as fraction of the year)? | 0.5 | Fixed number |  |  |  |  |  | No transformation |
| **59** | To what extent does movement of vectors contribute to long-distance spread in the area at risk? | *Mov_vector_* | Qualitative ranges | very low | low | moderate | high | very high | No transformation |
| **60** | To what extent does movement of hosts contribute to long-distance spread in the area at risk? | *Mov_host_* | Qualitative ranges | very low | low | moderate | high | very high | No transformation |
| **63** | What is the expected time (in years) until detection and reporting of the disease in the area at risk, leading to implementation of prevention and control measures if applicable? | *HRP_AaR_* | Quantitative ranges | < 0.1 | 0.1 – 0.3 | 0.3 – 1 | 1 – 3 | > 3 | ${10}^{\left( IV-0.6 \right)\times2.5}$ |
| **64** | What is the expected impact of control measures aiming at vector control and larval control on spread of the infection in the area at risk (achieved reduction of *R*)? | *CM_vector_* | Quantitative ranges | 1 | 1 – 2 | 2 – 4 | 4 – 8 | > 8 | If Answer=very low 1; Else $2^{\left( IV-0.2 \right)\times5}$ |
| **65** | What is the expected impact of control measures aiming at host animals on spread of the infection in the area at risk (achieved reduction of *R*)? | *CM_host_* | Quantitative ranges | 1 | 1 – 2 | 2 – 4 | 4 – 8 | > 8 | If Answer=very low 1; Else $2^{\left( IV-0.2 \right)\times5}$ |

| **Question number^a^** | **MINTRISK question** | **Input parameter for calculations** | **Answer type** | **Answer categories^b^** | | | | | **Log-transformation^c^** |
| --- | --- | --- | --- | --- | --- | --- | --- | --- | --- |
|  |  |  |  | Very low | Low | Moderate | High | Very high |  |
|  | Persistence |  |  |  |  |  |  |  |  |
| **73** | How likely is overwintering of infection in the area at risk via persistent infection of the host^h^ | *OW_host_PI_* | Quantitative ranges | < 10^-3^ | 10^-3^ – 0.01 | 0.01 – 0.1 | 0.1 – 0.95 | > 0.95 | If Answer=very high 1; Else ${10}^{\left( IV-0.8 \right)\times5}$ |
| **74** | How likely is overwintering of infection in the area at risk via vertical transmission in the host^h^ | *OW_host_VT_* | Quantitative ranges | < 10^-3^ | 10^-3^ – 0.01 | 0.01 – 0.1 | 0.1 – 0.95 | > 0.95 | If Answer=very high 1; Else ${10}^{\left( IV-0.8 \right)\times5}$ |
| **75** | How likely is overwintering of infection in the area at risk via direct host-to-host transmission^h^ | *OW_host_HT_* | Quantitative ranges | < 10^-3^ | 10^-3^ – 0.01 | 0.01 – 0.1 | 0.1 – 0.95 | > 0.95 | If Answer=very high 1; Else ${10}^{\left( IV-0.8 \right)\times5}$ |
| **77** | How likely is overwintering of infection in the area at risk via survival of an infected (adult) vector^h^ | *OW_vector_AD_* | Quantitative ranges | < 10^-3^ | 10^-3^ – 0.01 | 0.01 – 0.1 | 0.1 – 0.95 | > 0.95 | If Answer=very high 1; Else ${10}^{\left( IV-0.8 \right)\times5}$ |
| **78** | How likely is overwintering of infection in the area at risk via vertical transmission in the vector^h^ | *OW_vector_VT_* | Quantitative ranges | < 10^-3^ | 10^-3^ – 0.01 | 0.01 – 0.1 | 0.1 – 0.95 | > 0.95 | If Answer=very high 1; Else ${10}^{\left( IV-0.8 \right)\times5}$ |
| **80** | How likely is overwintering of infection in the area at risk via other mechanisms^h^ | *OW_other_* | Quantitative ranges | < 10^-3^ | 10^-3^ – 0.01 | 0.01 – 0.1 | 0.1 – 0.95 | > 0.95 | If Answer=very high 1; Else ${10}^{\left( IV-0.8 \right)\times5}$ |
|  | Economic impact |  |  |  |  |  |  |  |  |
| **86** | What are the expected direct agricultural economic losses per host (or herd/flock) (Euros)? | *Eco_DA_* | Quantitative ranges | < 10 | 10 - 100 | 100 - 10^3^ | 10^3^ – 10^4^ | > 10^4^ | ${10}^{IV\times5}$ |
| **87** | What are the expected indirect agricultural economic losses per host (or herd/flock) (Euros)? | *Eco_IA_* | Quantitative ranges | < 10 | 10 - 100 | 100 - 10^3^ | 10^3^ – 10^4^ | > 10^4^ | ${10}^{IV\times5}$ |

| **Question number^a^** | **MINTRISK question** | **Input parameter for calculations** | **Answer type** | **Answer categories^b^** | | | | | **Log-transformation^c^** |
| --- | --- | --- | --- | --- | --- | --- | --- | --- | --- |
|  |  |  |  | Very low | Low | Moderate | High | Very high |  |
| **84** | What are the expected indirect agricultural economic losses on national/regional level due to presence of the disease (independent of the number of hosts/herds/flocks infected), e.g. costs of movement stand-still and trade restrictions (Euros)? | *Eco_IC_* | Quantitative ranges | < 10^5^ | 10^5^ - 10^6^ | 10^6^ - 10^7^ | 10^7^ – 10^8^ | > 10^8^ | ${10}^{\left( IV+0.8 \right)\times5}$ |
| **85** | What are the expected economic losses due to human disease, per 100 animal hosts (Euros)?^g^ | *Eco_PH_* | Quantitative ranges | < 10^3^ | 10^3^ - 10^4^ | 10^4^ - 10^5^ | 10^5^ – 10^6^ | > 10^6^ | ${10}^{IV\times5}$ |
| **88** | What are the expected economic losses due to side effects (Euros)? | *Econ_SE_* | Quantitative ranges | < 10^3^ | 10^3^ - 10^4^ | 10^4^ - 10^5^ | 10^5^ – 10^6^ | > 10^6^ | ${10}^{\left( IV+0.4 \right)\times5}$ |
|  | Socio-ethical impact |  |  |  |  |  |  |  |  |
| **90** | How severe is the human disease burden (including impact due to anxiety)?^g^ | *Soc_PH_* | Qualitative | minimal | minor | moderate | major | massive | No transformation |
| **91** | How severe are consequences for animal welfare? | *Soc_AW_* | Qualitative | minimal | minor | moderate | major | massive | No transformation |
| **92** | To what extent do humans suffer due to disease in pet animals? | *Soc_PA_* | Qualitative | minimal | minor | moderate | major | massive | No transformation |
| **93** | To what extent is culling necessary to control the outbreak? | *Soc_CU_* | Qualitative | minimal | minor | moderate | major | massive | No transformation |
| **94** | To what extent do humans suffer due to loss of recreational outdoor space? | *Soc_RE_* | Qualitative | minimal | minor | moderate | major | massive | No transformation |

| **Question number^a^** | **MINTRISK question** | **Input parameter for calculations** | **Answer type** | **Answer categories^b^** | | | | | **Log-transformation^c^** |
| --- | --- | --- | --- | --- | --- | --- | --- | --- | --- |
|  |  |  |  | Very low | Low | Moderate | High | Very high |  |
|  | Environmental impact |  |  |  |  |  |  |  |  |
| **96** | How severe are consequences for biodiversity? | *Env_BD_* | Qualitative | minimal | minor | moderate | major | massive | No transformation |
| **97** | How severe are consequences for nature values? | *ENV_NV_* | Qualitative | minimal | minor | moderate | major | massive | No transformation |
| **98** | How severe are consequences of insecticides used to control vectors? | *ENV_VC_* | Qualitative | minimal | minor | moderate | major | massive | No transformation |

^a^ The numbering of questions in MINTRISK was based on the structured questionnaire of FEVER (Framework to assess Emerging VEctor-borne disease Risks) [1]. In FEVER, questions 1 to 17 are used for hazard identification. This step was not included in MINTRISK and therefore the first question in MINTRISK is question 18. Not all questions of the structured questionnaire of FEVER could be used for the semi-quantitative assessment resulting in missing question numbers in MINTRISK.

^b^ The five qualitative answer categories mostly range from very low to very high, but are sometimes named differently, e.g. from very short to very long or from minimal to massive.

^c^ The log-transformations are of the form $a^{\left( IV+b \right)\times c}$, where *IV* is the semi-quantitative input value obtained by Monte Carlo simulation (see Fig 2 of main paper), *a* is the base of the log-transformation (usually this is 10, but for parameters with a more narrow range, this can be 2 or 10^0.5^), *b* is a scaling parameter that determines the actual value of the parameter at which the risk score is 0, and *c* is the number of answer categories that fit within the risk score scale from 0 to 1 (always 5 in MINTRISK). Please note that for readability, log-transformations for parameters with *a* = 10^0.5^ were rewritten with *a* = 10 and *c* = 2.5.

^d^ If this question is answered with “No”, questions 19-27 do not need to be answered.

^e^ If this question is answered with “No”, questions 31-34 do not need to be answered.

^f^ These questions need to be addressed separately for each pathway and/or risk region.

^g^ These questions only need to be addressed if the infection is zoonotic.

^h^ For these questions, the very high answer category resembles a probability of (almost) 1; to ensure risk assessors would opt for this answer category in case of a very high probability, a narrow range was given for this answer category rather than a single value of 1.

**References**

1. De Vos C, Hoek M, Fischer E, De Koeijer A. Bremmer J. Risk assessment framework for emerging vector-borne livestock diseases. Report 11-CVI0168. Lelystad, the Netherlands: Central Veterinary Institute, part of Wageningen UR; 2011 [Cited 2018 Feb 15]. Available from: https://edepot.wur.nl/198115.
