# Supplementary material for: Assessing the introduction risk of vector-borne animal diseases for the Netherlands using MINTRISK: A Model for INTegrated RISK assessment
Source: PLoS One. 2021 Nov 2;16(11):e0259466. doi: 10.1371/journal.pone.0259466 (PMC8562800; doi:10.1371/journal.pone.0259466)
Supplement: S2 Appendix — (DOCX) [file pone.0259466.s002.docx]

**S2 Appendix: Overview of the intermediate results for each input step and results for each output parameter of MINTRISK.**

| **Input step / Output parameter** | **Parameter** | **Definition** | **Quantitative interpretation of risk levels** | | | | | **Inverse log-transformation^a^** |
| --- | --- | --- | --- | --- | --- | --- | --- | --- |
|  |  |  | Very low | Low | Moderate | High | Very high |  |
| **Rate of entry** | Entry | Expected number of infectious animals, contaminated commodities (in animal equivalents), infectious vectors, or infectious humans that enters the area at risk annually | < 0.01 | 0.01 – 0.1 | 0.1 – 1 | 1 – 10 | > 10 | $RS\_Entry=0.6+\frac{{Log}_{10}\left( Entry \right)}{5}$ |
| **Level of transmission** | *R_opt_* | The estimated R value under optimal conditions in the area at risk | < 0.3 | 0.3 – 1 | 1 – 3 | 3 – 10 | > 10 | $RS\_R_{opt}=0.4+\frac{{Log}_{10}\left( R_{opt} \right)}{2.5}$ |
| **Probability of establishment** | *Est* | Probability of successful establishment in the area at risk given entry of an infected pathway unit (i.e. an animal/vector/human/commodity) | < 10^-4^ | 10^-4^ – 10^-3^ | 10^-3^ – 0.01 | 0.01 – 0.1 | > 0.1 | $RS\_Est=1+\frac{{Log}_{10}\left( Est \right)}{5}$ |
| **Extent of spread** | *Inf_total_* | Number of infected host animals at the end of the vector season (or year) in which the disease was introduced | < 10 | 10 – 100 | 100 – 10^3^ | 10^3^ – 10^4^ | > 10^4^ | $RS\_{Inf}_{total}=\frac{{Log}_{10}\left( {Inf}_{total} \right)}{5}$ |
| **Persistence** | *Pers* | Expected number of infected hosts at the end of the adverse season that can initiate transmission at the start of the next vector season | < 0.01 | 0.01 – 0.1 | 0.1 – 1 | 1 – 10 | > 10 | $RS\_Pers=0.6+\frac{{Log}_{10}\left( Pers \right)}{5}$ |
| **Economic impact** | *Eco* | Expected economic losses (in euros) due to a single introduction of the disease, taking into account the expected transmission and persistence | < 10^5^ | 10^5^ – 10^6^ | 10^6^ – 10^7^ | 10^7^ – 10^8^ | > 10^8^ | $RS\_Eco=-0.8+\frac{{Log}_{10}\left( Eco \right)}{5}$ |
| **Rate of introduction** | *Intro* | Annual number of successful introductions in the area at risk by the pathway evaluated | < 10^-3^ | 10^-3^ – 0.01 | 0.01 – 0.1 | 0.1 – 1 | > 1 | $RS\_Intro=0.8+\frac{{Log}_{10}\left( Intro \right)}{5}$ |

| **Input step / Output parameter** | **Parameter** | **Definition** | **Quantitative interpretation of risk levels** | | | | | **Inverse log-transformation^a^** |
| --- | --- | --- | --- | --- | --- | --- | --- | --- |
|  |  |  | Very low | Low | Moderate | High | Very high |  |
| **Epidemic size** | *ES* | Total number of host animals infected after introduction of the disease, considering a maximum of four vector seasons (or years) | < 100 | 100 – 10^3^ | 10^3^ – 10^4^ | 10^4^ – 10^5^ | > 10^5^ | $RS\_ES=-0.2+\frac{{Log}_{10}\left( ES \right)}{5}$ |
| **Overall risk** | *Risk*^b^ | Expected economic losses (in euros) per year, considering the overall rate of introduction and the economic impact | < 10^3^ | 10^3^ – 10^4^ | 10^4^ – 10^5^ | 10^5^ – 10^6^ | > 10^6^ | $Risk=-0.4+{RS_{Intro}}_{final}+RS\_Eco$ |

^a^ *RS* = semi-quantitative risk score of the parameter. The inverse log-transformations are mostly of the form $a+\frac{{Log}_{b}\left( QV \right)}{c}$, where *QV* is the quantitative value of the parameter as calculated by MINTRISK, *a* is a scaling parameter that determines the quantitative value (*QV*) at which the risk score is 0, *b* is the base for the inverse log-transformation (usually this is 10, but it can also be 10^0.5^), and *c* is the number of risk score categories that fit within the semi-quantitative risk score scale from 0 to 1 (always 5 in MINTRISK). Please note that for readability, inverse log-transformations for parameters with *b* = 10^0.5^ were rewritten with *b* = 10 and *c* = 2.5.

^b^ This parameter is only available as a semi-quantitative risk score.
