# Supplementary material for: Assessing the introduction risk of vector-borne animal diseases for the Netherlands using MINTRISK: A Model for INTegrated RISK assessment
Source: PLoS One. 2021 Nov 2;16(11):e0259466. doi: 10.1371/journal.pone.0259466 (PMC8562800; doi:10.1371/journal.pone.0259466)
Supplement: S3 Appendix — (DOCX) [file pone.0259466.s003.docx]

**S3 Appendix: Overview of input in MINTRISK to assess the incursion risk of four vector-borne diseases to the Netherlands.**

**Table 1a. Overview of input in MINTRISK to assess the incursion risk of African horse sickness to the Netherlands: answer category chosen for each question with uncertainty level given in brackets (low, moderate, high) for the steps entry, transmission and establishment.**

| **Question number^a^** | **MINTRISK question** | **Introduction pathways** |  |  |  |  | **Sources^b^** |
| --- | --- | --- | --- | --- | --- | --- | --- |
|  |  | Illegal import of equines | Import of zoo animals | Midges via animal trade | Movement of competition horses | Import of modified live vaccines |  |
|  | Entry |  |  |  |  |  |  |
| **18** | Do epidemics of the disease occur somewhere in the risk region addressed? | Yes | Yes | Yes | Yes | Yes | 1,2 |
| **19** | What is relative size of the epidemic area related to the full risk region? | Moderate (mod) | Moderate (mod) | Moderate (mod) | Moderate (mod) | Moderate (mod) |  |
| **22** | What is the duration of the period (in years) between introduction of the infection into the region and notification of the infection (i.e. what is the length of the high risk period)? | Short (low) | Short (low) | Short (low) | Short (low) | Short (low) |  |
| **23** | Are humans considered dead-end hosts? | NA^c^ | NA | NA | NA | NA |  |
| **24** | What is the frequency (per year) with which epidemics occur in the risk region? | High (low) | High (low) | High (low) | High (low) | High (low) | 1 |
| **25** | How high is the prevalence of the infection in host animals in the risk region at the end of the high risk period? | Low (mod) | Low (mod) | Low (mod) | Low (mod) | Low (mod) | 1 |
| **26** | How high is the prevalence of the infection in vectors in the risk region at the end of the high risk period? | High (mod) | High (mod) | High (mod) | High (mod) | High (mod) | 3 |
| **27** | How high is the prevalence of the infection in humans in the risk region at the end of the high risk period? | NA | NA | NA | NA | NA |  |

| **Question number^a^** | **MINTRISK question** | **Introduction pathways** |  |  |  |  | **Sources^b^** |
| --- | --- | --- | --- | --- | --- | --- | --- |
|  |  | Illegal import of equines | Import of zoo animals | Midges via animal trade | Movement of competition horses | Import of modified live vaccines |  |
| **28** | Is disease endemic somewhere in the risk region addressed? | Yes | Yes | Yes | Yes | Yes | 1,2 |
| **31** | Are humans considered dead-end hosts? | NA | NA | NA | NA | NA |  |
| **32** | How high is the prevalence of the infection in host animals in the risk region? | Low (mod) | Low (mod) | Low (mod) | Low (mod) | Low (mod) | 1 |
| **33** | How high is the prevalence of the infection in vectors in the risk region? | Moderate (mod) | Moderate (mod) | Moderate (mod) | Moderate (mod) | Moderate (mod) | 3 |
| **34** | How high is the prevalence of the infection in humans in the risk region? | NA | NA | NA | NA | NA |  |
| **35** | What is the annual volume of animals / vectors / commodities / humans moved along the pathway from the risk region to the area at risk? | Minimal (mod) | Minimal (mod) | Minor (mod) | Minimal (mod) | Minimal (mod) | 4,5 |
| **39** | How likely is it that viable pathogen is still present in the animal, vector, commodity or human upon arrival in the area at risk? | High (low) | Very high (low) | Very high (low) | High (low) | Low (mod) |  |
| **41** | How likely is persistence / survival of infection in the animal, vector, commodity or human despite control / preventive measures during or after transport? | Very high (low) | Low (mod) | Very high (low) | Low (low) | Very high (low) | 6 |

| **Question number^a^** | **MINTRISK question** | **Introduction pathways** |  |  |  |  | **Sources^b^** |
| --- | --- | --- | --- | --- | --- | --- | --- |
|  |  | Illegal import of equines | Import of zoo animals | Midges via animal trade | Movement of competition horses | Import of modified live vaccines |  |
|  | Transmission |  |  |  |  |  |  |
| **45** | What is the distribution of the vector in the area at risk? | Homogeneous | Homogeneous | Homogeneous | Homogeneous | Homogeneous | 7 |
| **46** | What is the estimated value of the basic reproduction ratio? | Moderate (low) | Moderate (low) | Moderate (low) | Moderate (low) | Moderate (low) | 5,8,9 |
| **48** | Which fraction of the host population is susceptible to the infection (i.e. not protected from infection by routine vaccination or previous exposure)? | Very high (low) | Very high (low) | Very high (low) | Very high (low) | Very high (low) |  |
|  | Establishment |  |  |  |  |  |  |
| **50** | What is the probability of infecting a first local (indigenous) vector or host given the pathway of entry, and the expected region and time of entry? (first transmission step) | Very high (low) | Very high (low) | Very high (low) | Very high (low) | Very high (low) | 5,10,11 |
| **51** | What is the probability of infecting a first local vector (given first infection of an indigenous host) or host (given first infection of an indigenous vector)? (second transmission step) | High (low) | Very high (low) | Very high (low) | High (low) | Very high (low) | 5,10,11 |

^a^ The numbering of questions in MINTRISK was based on the structured questionnaire of FEVER (Framework to assess Emerging VEctor-borne disease Risks) [19]. In FEVER, questions 1 to 17 are used for hazard identification. This step was not included in MINTRISK and therefore the first question in MINTRISK is question 18. Not all questions of the structured questionnaire of FEVER could be used for the semi-quantitative assessment resulting in missing question numbers in MINTRISK

^b^ Sources used to evaluate the answers for the questions; if no source is mentioned, the answer is based on expert opinion

^c^ Question not applicable and therefore not answered

**Table 1b. Overview of input in MINTRISK to assess the incursion risk of African horse sickness to the Netherlands: answer category chosen for each question with uncertainty level given in brackets (low, moderate, high) for the steps spread, persistence and impact.**

| **Question number^a^** | **MINTRISK question** | **Answer category** | **Sources^b^** |
| --- | --- | --- | --- |
|  | Spread |  |  |
| **52** | What is the size of the (host) population at risk in the area at risk? | Moderate (low) | 12,13 |
| **53** | What is the expected number of infection generations per vector season? | 6 |  |
| **54** | What is the overlap between (high) vector abundance and host density in the area at risk? | Very high (low) | 7 |
| **55** | To what extent does the presence of non-susceptible hosts in the area at risk result in a dilution effect? | Totally (low) | 11,14 |
| **56** | To what extent is local spread in the area at risk inhibited by spatial effects? | Little (low) |  |
| **57** | What is the expected length of the vector season (expressed as fraction of the year)? | 0.5 |  |
| **59** | To what extent does movement of vectors contribute to long-distance spread in the area at risk? | High (mod) |  |
| **60** | To what extent does movement of hosts contribute to long-distance spread in the area at risk? | Very high (low) |  |
| **63** | What is the expected time (in years) until detection and reporting of the disease in the area at risk, leading to implementation of prevention and control measures if applicable? | Short (low) | 5 |
| **64** | What is the expected impact of control measures aiming at vector control and larval control on spread of the infection in the area at risk (achieved reduction of *R*)? | Low (mod) |  |

| **Question number^a^** | **MINTRISK question** | **Answer category** | **Sources^b^** |
| --- | --- | --- | --- |
| **65** | What is the expected impact of control measures aiming at host animals on spread of the infection in the area at risk (achieved reduction of *R*)? | Low (mod) |  |
|  | Persistence |  |  |
| **73** | How likely is overwintering of infection in the area at risk via persistent infection of the host | Very low (low) | 2,15,16 |
| **74** | How likely is overwintering of infection in the area at risk via vertical transmission in the host | Very low (low) |  |
| **75** | How likely is overwintering of infection in the area at risk via direct host-to-host transmission | Very low (low) |  |
| **77** | How likely is overwintering of infection in the area at risk via survival of an infected (adult) vector | Low (mod) |  |
| **78** | How likely is overwintering of infection in the area at risk via vertical transmission in the vector | Very low (low) | 17 |
| **80** | How likely is overwintering of infection in the area at risk via other mechanisms | Low (mod) | 18 |
|  | Economic impact |  |  |
| **86** | What are the expected direct agricultural economic losses per host (or herd/flock) (Euros)? | Massive (mod) | 13 |
| **87** | What are the expected indirect agricultural economic losses per host (or herd/flock) (Euros)? | Moderate (high) | 13 |
| **84** | What are the expected indirect agricultural economic losses on national/regional level due to presence of the disease (independent of the number of hosts/herds/flocks infected), e.g. costs of movement stand-still and trade restrictions (Euros)? | Massive (low) | 13 |
| **85** | What are the expected economic losses due to human disease, per 100 animal hosts (Euros)? | NA^c^ |  |
| **Question number^a^** | **MINTRISK question** | **Answer category** | **Sources^b^** |
| **88** | What are the expected economic losses due to side effects (Euros)? | Major (mod) |  |
|  | Socio-ethical impact |  |  |
| **90** | How severe is the human disease burden (including impact due to anxiety)? | NA |  |
| **91** | How severe are consequences for animal welfare? | Massive (low) |  |
| **92** | To what extent do humans suffer due to disease in pet animals? | Massive (low) |  |
| **93** | To what extent is culling necessary to control the outbreak? | Major (mod) |  |
| **94** | To what extent do humans suffer due to loss of recreational outdoor space? | Minor (mod) |  |
|  | Environmental impact |  |  |
| **96** | How severe are consequences for biodiversity? | Absent^d^ |  |
| **97** | How severe are consequences for nature values? | Minimal (low) |  |
| **98** | How severe are consequences of insecticides used to control vectors? | Minimal (low) |  |

^a^ The numbering of questions in MINTRISK was based on the structured questionnaire of FEVER (Framework to assess Emerging VEctor-borne disease Risks) [19]. In FEVER, questions 1 to 17 are used for hazard identification. This step was not included in MINTRISK and therefore the first question in MINTRISK is question 18. Not all questions of the structured questionnaire of FEVER could be used for the semi-quantitative assessment resulting in missing question numbers in MINTRISK.

^b^ Sources used to evaluate the answers for the questions; if no source is mentioned, the answer is based on expert opinion.

^c^ Question not applicable and therefore not answered.

^d^ Questions to assess economic, socio-ethical or environmental impact can also be answered with ‘absent’ if no impact is expected at all.

**Table 2a. Overview of input in MINTRISK to assess the incursion risk of epizootic haemorrhagic disease to the Netherlands: answer category chosen for each question with uncertainty level given in brackets (low, moderate, high) for the steps entry, transmission and establishment.**

| **Question number^a^** | **MINTRISK question** | **Introduction pathways** |  |  |  |  |  | **Sources^b^** |
| --- | --- | --- | --- | --- | --- | --- | --- | --- |
|  |  | Illegal import of livestock from Mediterranean countries | Midges via animal trade from Mediterranean countries | Midges via road transport from Mediterranean countries | Import of biological materials from Mediterranean countries | Midges via animal trade from the USA or Australia | Import of biological materials from the USA or Australia |  |
|  | Entry |  |  |  |  |  |  |  |
| **18** | Do epidemics of the disease occur somewhere in the risk region addressed? | Yes | Yes | Yes | Yes | No | No |  |
| **19** | What is relative size of the epidemic area related to the full risk region? | Moderate (mod) | Moderate (mod) | Moderate (mod) | Moderate (mod) | NA | NA | 20 |
| **22** | What is the duration of the period (in years) between introduction of the infection into the region and notification of the infection (i.e. what is the length of the high risk period)? | Moderate (mod) | Moderate (mod) | Moderate (mod) | Moderate (mod) | NA | NA |  |
| **23** | Are humans considered dead-end hosts? | NA^c^ | NA | NA | NA | NA | NA |  |
| **24** | What is the frequency (per year) with which epidemics occur in the risk region? | Moderate (low) | Moderate (low) | Moderate (low) | Moderate (low) | NA | NA | 1 |
| **25** | How high is the prevalence of the infection in host animals in the risk region at the end of the high risk period? | High (mod) | High (mod) | High (mod) | High (mod) | NA | NA | 18 |
| **26** | How high is the prevalence of the infection in vectors in the risk region at the end of the high risk period? | High (mod) | High (mod) | High (mod) | High (mod) | NA | NA | 21,22 |
| **27** | How high is the prevalence of the infection in humans in the risk region at the end of the high risk period? | NA | NA | NA | NA | NA | NA |  |

| **Question number^a^** | **MINTRISK question** | **Introduction pathways** |  |  |  |  |  | **Sources^b^** |
| --- | --- | --- | --- | --- | --- | --- | --- | --- |
|  |  | Illegal import of livestock from Mediterranean countries | Midges via animal trade from Mediterranean countries | Midges via road transport from Mediterranean countries | Import of biological materials from Mediterranean countries | Midges via animal trade from the USA or Australia | Import of biological materials from the USA or Australia |  |
| **28** | Is disease endemic somewhere in the risk region addressed? | No | No | No | No | Yes | Yes |  |
| **31** | Are humans considered dead-end hosts? | NA | NA | NA | NA | NA | NA |  |
| **32** | How high is the prevalence of the infection in host animals in the risk region? | NA | NA | NA | NA | Moderate (mod) | Moderate (mod) | 18 |
| **33** | How high is the prevalence of the infection in vectors in the risk region? | NA | NA | NA | NA | Very low (mod) | Very low (mod) | 21,22 |
| **34** | How high is the prevalence of the infection in humans in the risk region? | NA | NA | NA | NA | NA | NA |  |
| **35** | What is the annual volume of animals / vectors / commodities / humans moved along the pathway from the risk region to the area at risk? | Minimal (mod) | Minor (mod) | Minor (mod) | Minimal (mod) | Minor (mod) | Minor (mod) | 4,5,6 |
| **39** | How likely is it that viable pathogen is still present in the animal, vector, commodity or human upon arrival in the area at risk? | Very high (low) | Very high (low) | Very high (low) | Very high (low) | Very high (low) | Very high (low) | 20 |
| **41** | How likely is persistence / survival of infection in the animal, vector, commodity or human despite control / preventive measures during or after transport? | High (low) | Very high (low) | Very high (low) | Very high (low) | Very high (low) | Very high (low) |  |

| **Question number^a^** | **MINTRISK question** | **Introduction pathways** |  |  |  |  |  | **Sources^b^** |
| --- | --- | --- | --- | --- | --- | --- | --- | --- |
|  |  | Illegal import of livestock from Mediterranean countries | Midges via animal trade from Mediterranean countries | Midges via road transport from Mediterranean countries | Import of biological materials from Mediterranean countries | Midges via animal trade from the USA or Australia | Import of biological materials from the USA or Australia |  |
|  | Transmission |  |  |  |  |  |  |  |
| **45** | What is the distribution of the vector in the area at risk? | Homogeneous | Homogeneous | Homogeneous | Homogeneous | Homogeneous | Homogeneous | 7 |
| **46** | What is the estimated value of the basic reproduction ratio? | Moderate (mod) | Moderate (mod) | Moderate (mod) | Moderate (mod) | Moderate (mod) | Moderate (mod) | 23,24 |
| **48** | Which fraction of the host population is susceptible to the infection (i.e. not protected from infection by routine vaccination or previous exposure)? | Very high (low) | Very high (low) | Very high (low) | Very high (low) | Very high (low) | Very high (low) |  |
|  | Establishment |  |  |  |  |  |  |  |
| **50** | What is the probability of infecting a first local (indigenous) vector or host given the pathway of entry, and the expected region and time of entry? (first transmission step) | Very high (low) | Very high (low) | High (mod) | Low (low) | Very high (low) | Low (low) | 5,10,11 |
| **51** | What is the probability of infecting a first local vector (given first infection of an indigenous host) or host (given first infection of an indigenous vector)? (second transmission step) | Very high (low) | Very high (low) | Very high (low) | Very high (low) | Very high (low) | Very high (low) | 5,10,11 |

^a^ The numbering of questions in MINTRISK was based on the structured questionnaire of FEVER (Framework to assess Emerging VEctor-borne disease Risks) [19]. In FEVER, questions 1 to 17 are used for hazard identification. This step was not included in MINTRISK and therefore the first question in MINTRISK is question 18. Not all questions of the structured questionnaire of FEVER could be used for the semi-quantitative assessment resulting in missing question numbers in MINTRISK

^b^ Sources used to evaluate the answers for the questions; if no source is mentioned, the answer is based on expert opinion

^c^ Question not applicable and therefore not answered

**Table 2b. Overview of input in MINTRISK to assess the incursion risk of epizootic haemorrhagic disease to the Netherlands: answer category chosen for each question with uncertainty level given in brackets (low, moderate, high) for the steps spread, persistence and impact.**

| **Question number^a^** | **MINTRISK question** | **Answer category** | **Sources^b^** |
| --- | --- | --- | --- |
|  | Spread |  |  |
| **52** | What is the size of the (host) population at risk in the area at risk? | Large (low) | 14 |
| **53** | What is the expected number of infection generations per vector season? | 6 |  |
| **54** | What is the overlap between (high) vector abundance and host density in the area at risk? | Very high (low) | 7,14 |
| **55** | To what extent does the presence of non-susceptible hosts in the area at risk result in a dilution effect? | Minimal (low) | 10,14 |
| **56** | To what extent is local spread in the area at risk inhibited by spatial effects? | Little (low) |  |
| **57** | What is the expected length of the vector season (expressed as fraction of the year)? | 0.5 |  |
| **59** | To what extent does movement of vectors contribute to long-distance spread in the area at risk? | High (mod) |  |
| **60** | To what extent does movement of hosts contribute to long-distance spread in the area at risk? | Very high (low) |  |
| **63** | What is the expected time (in years) until detection and reporting of the disease in the area at risk, leading to implementation of prevention and control measures if applicable? | Moderate (mod) |  |
| **64** | What is the expected impact of control measures aiming at vector control and larval control on spread of the infection in the area at risk (achieved reduction of *R*)? | Low (mod) |  |

| **Question number^a^** | **MINTRISK question** | **Answer category** | **Sources^b^** |
| --- | --- | --- | --- |
| **65** | What is the expected impact of control measures aiming at host animals on spread of the infection in the area at risk (achieved reduction of *R*)? | Low (mod) |  |
|  | Persistence |  |  |
| **73** | How likely is overwintering of infection in the area at risk via persistent infection of the host | Very low (low) | 15,20 |
| **74** | How likely is overwintering of infection in the area at risk via vertical transmission in the host | Moderate (mod) | 20,25 |
| **75** | How likely is overwintering of infection in the area at risk via direct host-to-host transmission | Very low (mod) |  |
| **77** | How likely is overwintering of infection in the area at risk via survival of an infected (adult) vector | Moderate (mod) |  |
| **78** | How likely is overwintering of infection in the area at risk via vertical transmission in the vector | Very low (low) | 17 |
| **80** | How likely is overwintering of infection in the area at risk via other mechanisms | Moderate (mod) | 18,26 |
|  | Economic impact |  |  |
| **86** | What are the expected direct agricultural economic losses per host (or herd/flock) (Euros)? | Major (mod) | 27,28 |
| **87** | What are the expected indirect agricultural economic losses per host (or herd/flock) (Euros)? | Moderate (high) |  |
| **84** | What are the expected indirect agricultural economic losses on national/regional level due to presence of the disease (independent of the number of hosts/herds/flocks infected), e.g. costs of movement stand-still and trade restrictions (Euros)? | Major (mod) | 28 |
| **85** | What are the expected economic losses due to human disease, per 100 animal hosts (Euros)? | NA^c^ |  |

| **Question number^a^** | **MINTRISK question** | **Answer category** | **Sources^b^** |
| --- | --- | --- | --- |
| **88** | What are the expected economic losses due to side effects (Euros)? | Minor (mod) |  |
|  | Socio-ethical impact |  |  |
| **90** | How severe is the human disease burden (including impact due to anxiety)? | NA |  |
| **91** | How severe are consequences for animal welfare? | Moderate (mod) |  |
| **92** | To what extent do humans suffer due to disease in pet animals? | Minimal (low) |  |
| **93** | To what extent is culling necessary to control the outbreak? | Minimal (mod) |  |
| **94** | To what extent do humans suffer due to loss of recreational outdoor space? | Minor (mod) |  |
|  | Environmental impact |  |  |
| **96** | How severe are consequences for biodiversity? | Moderate (high) | 20,29 |
| **97** | How severe are consequences for nature values? | Minimal (low) |  |
| **98** | How severe are consequences of insecticides used to control vectors? | Minimal (low) |  |

^a^ The numbering of questions in MINTRISK was based on the structured questionnaire of FEVER (Framework to assess Emerging VEctor-borne disease Risks) [19]. In FEVER, questions 1 to 17 are used for hazard identification. This step was not included in MINTRISK and therefore the first question in MINTRISK is question 18. Not all questions of the structured questionnaire of FEVER could be used for the semi-quantitative assessment resulting in missing question numbers in MINTRISK.

^b^ Sources used to evaluate the answers for the questions; if no source is mentioned, the answer is based on expert opinion.

^c^ Question not applicable and therefore not answered.

**Table 3a. Overview of input in MINTRISK to assess the incursion risk of Rift Valley fever to the Netherlands: answer category chosen for each question with uncertainty level given in brackets (low, moderate, high) for the steps entry, transmission and establishment.**

| **Question number^a^** | **MINTRISK question** | **Introduction pathways** |  |  |  |  | **Sources^b^** |
| --- | --- | --- | --- | --- | --- | --- | --- |
|  |  | Infected mosquitoes via containers | Infected mosquitoes via aircraft | Import of live animals (illegal) | Import of live animals (legal) | Infected mosquito eggs via plants or tires |  |
|  | Entry |  |  |  |  |  |  |
| **18** | Do epidemics of the disease occur somewhere in the risk region addressed? | Yes | Yes | Yes | Yes | Yes | 1,30 |
| **19** | What is relative size of the epidemic area related to the full risk region? | Moderate (low) | Moderate (low) | Moderate (low) | Moderate (low) | Moderate (low) |  |
| **22** | What is the duration of the period (in years) between introduction of the infection into the region and notification of the infection (i.e. what is the length of the high risk period)? | Moderate (mod) | Moderate (mod) | Moderate (mod) | Moderate (mod) | Moderate (mod) |  |
| **23** | Are humans considered dead-end hosts? | Yes | Yes | Yes | Yes | Yes |  |
| **24** | What is the frequency (per year) with which epidemics occur in the risk region? | High (low) | High (low) | High (low) | High (low) | High (low) | 1 |
| **25** | How high is the prevalence of the infection in host animals in the risk region at the end of the high risk period? | High (mod) | High (mod) | High (mod) | High (mod) | High (mod) | 31,32,33 |
| **26** | How high is the prevalence of the infection in vectors in the risk region at the end of the high risk period? | High (mod) | High (mod) | High (mod) | High (mod) | High (mod) | 31 |
| **27** | How high is the prevalence of the infection in humans in the risk region at the end of the high risk period? | NA^c^ | NA | NA | NA | NA |  |

| **Question number^a^** | **MINTRISK question** | **Introduction pathways** |  |  |  |  | **Sources^b^** |
| --- | --- | --- | --- | --- | --- | --- | --- |
|  |  | Infected mosquitoes via containers | Infected mosquitoes via aircraft | Import of live animals (illegal) | Import of live animals (legal) | Infected mosquito eggs via plants or tires |  |
| **28** | Is disease endemic somewhere in the risk region addressed? | Yes | Yes | Yes | No | Yes | 1,30 |
| **31** | Are humans considered dead-end hosts? | Yes | Yes | Yes | NA | Yes |  |
| **32** | How high is the prevalence of the infection in host animals in the risk region? | High (mod) | High (mod) | High (mod) | NA | High (mod) | 34 |
| **33** | How high is the prevalence of the infection in vectors in the risk region? | Moderate (mod) | Moderate (mod) | Moderate (mod) | NA | Moderate (mod) | 31 |
| **34** | How high is the prevalence of the infection in humans in the risk region? | NA | NA | NA | NA | NA |  |
| **35** | What is the annual volume of animals / vectors / commodities / humans moved along the pathway from the risk region to the area at risk? | Minor (high) | Moderate (mod) | Minimal (mod) | Minimal (low) | Major (high) | 35,36,37,38,39 |
| **39** | How likely is it that viable pathogen is still present in the animal, vector, commodity or human upon arrival in the area at risk? | Very high (mod) | Very high (low) | High (mod) | High (mod) | Low (low) | 40,41,42 |
| **41** | How likely is persistence / survival of infection in the animal, vector, commodity or human despite control / preventive measures during or after transport? | Very high (low) | Very high (low) | Very high (low) | Moderate (mod) | Very high (mod) |  |

| **Question number^a^** | **MINTRISK question** | **Introduction pathways** |  |  |  |  | **Sources^b^** |
| --- | --- | --- | --- | --- | --- | --- | --- |
|  |  | Infected mosquitoes via containers | Infected mosquitoes via aircraft | Import of live animals (illegal) | Import of live animals (legal) | Infected mosquito eggs via plants or tires |  |
|  | Transmission |  |  |  |  |  |  |
| **45** | What is the distribution of the vector in the area at risk? | Homogeneous | Homogeneous | Homogeneous | Homogeneous | Homogeneous | 41,43 |
| **46** | What is the estimated value of the basic reproduction ratio? | Moderate (mod) | Moderate (mod) | Moderate (mod) | Moderate (mod) | Moderate (mod) | 9,15,41 |
| **48** | Which fraction of the host population is susceptible to the infection (i.e. not protected from infection by routine vaccination or previous exposure)? | Very high (low) | Very high (low) | Very high (low) | Very high (low) | Very high (low) |  |
|  | Establishment |  |  |  |  |  |  |
| **50** | What is the probability of infecting a first local (indigenous) vector or host given the pathway of entry, and the expected region and time of entry? (first transmission step) | High (mod) | Moderate (mod) | Very high (mod) | Very high (mod) | Mod (high) | 41 |
| **51** | What is the probability of infecting a first local vector (given first infection of an indigenous host) or host (given first infection of an indigenous vector)? (second transmission step) | Very high (low) | Very high (low) | High (mod) | High (mod) | Very high (low) | 41 |

^a^ The numbering of questions in MINTRISK was based on the structured questionnaire of FEVER (Framework to assess Emerging VEctor-borne disease Risks) [19]. In FEVER, questions 1 to 17 are used for hazard identification. This step was not included in MINTRISK and therefore the first question in MINTRISK is question 18. Not all questions of the structured questionnaire of FEVER could be used for the semi-quantitative assessment resulting in missing question numbers in MINTRISK.

^b^ Sources used to evaluate the answers for the questions; if no source is mentioned, the answer is based on expert opinion.

^c^ Question not applicable and therefore not answered.

**Table 3b. Overview of input in MINTRISK to assess the incursion risk of Rift Valley fever to the Netherlands: answer category chosen for each question with uncertainty level given in brackets (low, moderate, high) for the steps spread, persistence and impact.**

| **Question number^a^** | **MINTRISK question** | **Answer category** | **Sources^b^** |
| --- | --- | --- | --- |
|  | Spread |  |  |
| **52** | What is the size of the (host) population at risk in the area at risk? | Large (low) | 14 |
| **53** | What is the expected number of infection generations per vector season? | 6 |  |
| **54** | What is the overlap between (high) vector abundance and host density in the area at risk? | Very high (low) | 41 |
| **55** | To what extent does the presence of non-susceptible hosts in the area at risk result in a dilution effect? | Totally (mod) |  |
| **56** | To what extent is local spread in the area at risk inhibited by spatial effects? | Moderate (low) |  |
| **57** | What is the expected length of the vector season (expressed as fraction of the year)? | 0.5 |  |
| **59** | To what extent does movement of vectors contribute to long-distance spread in the area at risk? | Very low (mod) | 44 |
| **60** | To what extent does movement of hosts contribute to long-distance spread in the area at risk? | Very high (low) |  |
| **63** | What is the expected time (in years) until detection and reporting of the disease in the area at risk, leading to implementation of prevention and control measures if applicable? | Long (low) |  |
| **64** | What is the expected impact of control measures aiming at vector control and larval control on spread of the infection in the area at risk (achieved reduction of *R*)? | Very low (low) |  |

| **Question number^a^** | **MINTRISK question** | **Answer category** | **Sources^b^** |
| --- | --- | --- | --- |
| **65** | What is the expected impact of control measures aiming at host animals on spread of the infection in the area at risk (achieved reduction of *R*)? | Moderate (mod) |  |
|  | Persistence |  |  |
| **73** | How likely is overwintering of infection in the area at risk via persistent infection of the host | Very low (low) | 31,42 |
| **74** | How likely is overwintering of infection in the area at risk via vertical transmission in the host | High (mod) | 42,45 |
| **75** | How likely is overwintering of infection in the area at risk via direct host-to-host transmission | Low (mod) | 46 |
| **77** | How likely is overwintering of infection in the area at risk via survival of an infected (adult) vector | Moderate (mod) | 47,48,49 |
| **78** | How likely is overwintering of infection in the area at risk via vertical transmission in the vector | Moderate (high) | 31,40,41,50 |
| **80** | How likely is overwintering of infection in the area at risk via other mechanisms | Low (mod) | 44 |
|  | Economic impact |  |  |
| **86** | What are the expected direct agricultural economic losses per host (or herd/flock) (Euros)? | Moderate (mod) | 51,52 |
| **87** | What are the expected indirect agricultural economic losses per host (or herd/flock) (Euros)? | Minor (mod) | 51 |
| **84** | What are the expected indirect agricultural economic losses on national/regional level due to presence of the disease (independent of the number of hosts/herds/flocks infected), e.g. costs of movement stand-still and trade restrictions (Euros)? | Massive (low) | 53 |
| **85** | What are the expected economic losses due to human disease, per 100 animal hosts (Euros)? | Moderate (mod) | 51 |

| **Question number^a^** | **MINTRISK question** | **Answer category** | **Sources^b^** |
| --- | --- | --- | --- |
| **88** | What are the expected economic losses due to side effects (Euros)? | Massive (mod) | 53 |
|  | Socio-ethical impact |  |  |
| **90** | How severe is the human disease burden (including impact due to anxiety)? | Massive (mod) |  |
| **91** | How severe are consequences for animal welfare? | Major (mod) |  |
| **92** | To what extent do humans suffer due to disease in pet animals? | Minimal (low) |  |
| **93** | To what extent is culling necessary to control the outbreak? | Minimal (mod) |  |
| **94** | To what extent do humans suffer due to loss of recreational outdoor space? | Minor (mod) |  |
|  | Environmental impact |  |  |
| **96** | How severe are consequences for biodiversity? | Minimal (low) |  |
| **97** | How severe are consequences for nature values? | Minimal (low) |  |
| **98** | How severe are consequences of insecticides used to control vectors? | Minimal (low) |  |

^a^ The numbering of questions in MINTRISK was based on the structured questionnaire of FEVER (Framework to assess Emerging VEctor-borne disease Risks) [19]. In FEVER, questions 1 to 17 are used for hazard identification. This step was not included in MINTRISK and therefore the first question in MINTRISK is question 18. Not all questions of the structured questionnaire of FEVER could be used for the semi-quantitative assessment resulting in missing question numbers in MINTRISK.

^b^ Sources used to evaluate the answers for the questions; if no source is mentioned, the answer is based on expert opinion.

**Table 4a. Overview of input in MINTRISK to assess the incursion risk of West Nile fever to the Netherlands: answer category chosen for each question with uncertainty level given in brackets (low, moderate, high) for the steps entry, transmission and establishment.**

| **Question number^a^** | **MINTRISK question** | **Introduction pathways** |  |  | **Sources^b^** |
| --- | --- | --- | --- | --- | --- |
|  |  | Infected mosquitoes via containers | Infected mosquitoes via aircraft | Migratory birds |  |
|  | Entry |  |  |  |  |
| **18** | Do epidemics of the disease occur somewhere in the risk region addressed? | No | No | No |  |
| **19** | What is relative size of the epidemic area related to the full risk region? | NA^c^ | NA | NA |  |
| **22** | What is the duration of the period (in years) between introduction of the infection into the region and notification of the infection (i.e. what is the length of the high risk period)? | NA | NA | NA |  |
| **23** | Are humans considered dead-end hosts? | NA | NA | NA |  |
| **24** | What is the frequency (per year) with which epidemics occur in the risk region? | NA | NA | NA |  |
| **25** | How high is the prevalence of the infection in host animals in the risk region at the end of the high risk period? | NA | NA | NA |  |
| **26** | How high is the prevalence of the infection in vectors in the risk region at the end of the high risk period? | NA | NA | NA |  |
| **27** | How high is the prevalence of the infection in humans in the risk region at the end of the high risk period? | NA | NA | NA |  |

| **Question number^a^** | **MINTRISK question** | **Introduction pathways** |  |  | **Sources^b^** |
| --- | --- | --- | --- | --- | --- |
|  |  | Infected mosquitoes via containers | Infected mosquitoes via aircraft | Migratory birds |  |
| **28** | Is disease endemic somewhere in the risk region addressed? | Yes | Yes | Yes | 54 |
| **31** | Are humans considered dead-end hosts? | Yes | Yes | Yes |  |
| **32** | How high is the prevalence of the infection in host animals in the risk region? | Moderate (mod) | Moderate (mod) | Moderate (mod) | 55,56,57,58,59,60,61 |
| **33** | How high is the prevalence of the infection in vectors in the risk region? | Moderate (mod) | Moderate (mod) | Moderate (mod) | 36,59,60,62,63,64 |
| **34** | How high is the prevalence of the infection in humans in the risk region? | NA | NA | NA |  |
| **35** | What is the annual volume of animals / vectors / commodities / humans moved along the pathway from the risk region to the area at risk? | Moderate (high) | Major (mod) | Massive (low) | 35,36,38,39 |
| **39** | How likely is it that viable pathogen is still present in the animal, vector, commodity or human upon arrival in the area at risk? | Very high (mod) | Very high (low) | Low (high) | 65 |
| **41** | How likely is persistence / survival of infection in the animal, vector, commodity or human despite control / preventive measures during or after transport? | Very high (low) | Very high (low) | Very high (low) |  |
|  | Transmission |  |  |  |  |
| **45** | What is the distribution of the vector in the area at risk? | Homogeneous | Homogeneous | Homogeneous | 41,43 |
| **46** | What is the estimated value of the basic reproduction ratio? | High (mod) | High (mod) | High (mod) | 9,66,67,68,69,70 |
| **48** | Which fraction of the host population is susceptible to the infection (i.e. not protected from infection by routine vaccination or previous exposure)? | Very high (low) | Very high (low) | Very high (low) |  |

| **Question number^a^** | **MINTRISK question** | **Introduction pathways** |  |  | **Sources^b^** |
| --- | --- | --- | --- | --- | --- |
|  |  | Infected mosquitoes via containers | Infected mosquitoes via aircraft | Migratory birds |  |
|  | Establishment |  |  |  |  |
| **50** | What is the probability of infecting a first local (indigenous) vector or host given the pathway of entry, and the expected region and time of entry? (first transmission step) | Very high (low) | Very high (low) | Very high (mod) | 70,71 |
| **51** | What is the probability of infecting a first local vector (given first infection of an indigenous host) or host (given first infection of an indigenous vector)? (second transmission step) | Very high (mod) | Very high (mod) | Very high (mod) | 70,71 |

^a^ The numbering of questions in MINTRISK was based on the structured questionnaire of FEVER (Framework to assess Emerging VEctor-borne disease Risks) [19]. In FEVER, questions 1 to 17 are used for hazard identification. This step was not included in MINTRISK and therefore the first question in MINTRISK is question 18. Not all questions of the structured questionnaire of FEVER could be used for the semi-quantitative assessment resulting in missing question numbers in MINTRISK.

^b^ Sources used to evaluate the answers for the questions; if no source is mentioned, the answer is based on expert opinion.

^c^ Question not applicable and therefore not answered.

**Table 4b. Overview of input in MINTRISK to assess the incursion risk of West Nile fever to the Netherlands: answer category chosen for each question with uncertainty level given in brackets (low, moderate, high) for the steps spread, persistence and impact.**

| **Question number^a^** | **MINTRISK question** | **Answer category** | **Sources^b^** |
| --- | --- | --- | --- |
|  | Spread |  |  |
| **52** | What is the size of the (host) population at risk in the area at risk? | Very large (low) | 65,72,73 |
| **53** | What is the expected number of infection generations per vector season? | 6 |  |
| **54** | What is the overlap between (high) vector abundance and host density in the area at risk? | Very high (low) | 41,43,73 |
| **55** | To what extent does the presence of non-susceptible hosts in the area at risk result in a dilution effect? | Largely (mod) | 65,72 |
| **56** | To what extent is local spread in the area at risk inhibited by spatial effects? | Little (mod) |  |
| **57** | What is the expected length of the vector season (expressed as fraction of the year)? | 0.5 |  |
| **59** | To what extent does movement of vectors contribute to long-distance spread in the area at risk? | Very low (mod) | 44 |
| **60** | To what extent does movement of hosts contribute to long-distance spread in the area at risk? | Moderate (mod) |  |
| **63** | What is the expected time (in years) until detection and reporting of the disease in the area at risk, leading to implementation of prevention and control measures if applicable? | Moderate (mod) |  |
| **64** | What is the expected impact of control measures aiming at vector control and larval control on spread of the infection in the area at risk (achieved reduction of *R*)? | Very low (low) |  |

| **Question number^a^** | **MINTRISK question** | **Introduction pathways** | **Sources^b^** |
| --- | --- | --- | --- |
| **65** | What is the expected impact of control measures aiming at host animals on spread of the infection in the area at risk (achieved reduction of *R*)? | Very low (low) |  |
|  | Persistence |  |  |
| **73** | How likely is overwintering of infection in the area at risk via persistent infection of the host | Very low (mod) | 15,72 |
| **74** | How likely is overwintering of infection in the area at risk via vertical transmission in the host | Very low (low) |  |
| **75** | How likely is overwintering of infection in the area at risk via direct host-to-host transmission | Moderate (mod) | 74 |
| **77** | How likely is overwintering of infection in the area at risk via survival of an infected (adult) vector | Moderate (mod) | 47,48,49,75,76 |
| **78** | How likely is overwintering of infection in the area at risk via vertical transmission in the vector | Low (mod) | 77,78,79,80 |
| **80** | How likely is overwintering of infection in the area at risk via other mechanisms | Low (mod) | 65 |
|  | Economic impact |  |  |
| **86** | What are the expected direct agricultural economic losses per host (or herd/flock) (Euros)? | Minimal (low) | 65,81 |
| **87** | What are the expected indirect agricultural economic losses per host (or herd/flock) (Euros)? | Minimal (low) | 65,81 |
| **84** | What are the expected indirect agricultural economic losses on national/regional level due to presence of the disease (independent of the number of hosts/herds/flocks infected), e.g. costs of movement stand-still and trade restrictions (Euros)? | Minimal (low) |  |
| **85** | What are the expected economic losses due to human disease, per 100 animal hosts (Euros)? | Minimal (low) | 65,82 |

| **Question number^a^** | **MINTRISK question** | **Introduction pathways** | **Sources^b^** |
| --- | --- | --- | --- |
| **88** | What are the expected economic losses due to side effects (Euros)? | Minor (mod) |  |
|  | Socio-ethical impact |  |  |
| **90** | How severe is the human disease burden (including impact due to anxiety)? | Major (high) |  |
| **91** | How severe are consequences for animal welfare? | Moderate (mod) |  |
| **92** | To what extent do humans suffer due to disease in pet animals? | Major (mod) |  |
| **93** | To what extent is culling necessary to control the outbreak? | Absent^c^ |  |
| **94** | To what extent do humans suffer due to loss of recreational outdoor space? | Minor (mod) |  |
|  | Environmental impact |  |  |
| **96** | How severe are consequences for biodiversity? | Moderate (mod) | 72 |
| **97** | How severe are consequences for nature values? | Minimal (low) |  |
| **98** | How severe are consequences of insecticides used to control vectors? | Minimal (low) |  |

^a^ The numbering of questions in MINTRISK was based on the structured questionnaire of FEVER (Framework to assess Emerging VEctor-borne disease Risks) [19]. In FEVER, questions 1 to 17 are used for hazard identification. This step was not included in MINTRISK and therefore the first question in MINTRISK is question 18. Not all questions of the structured questionnaire of FEVER could be used for the semi-quantitative assessment resulting in missing question numbers in MINTRISK.

^b^ Sources used to evaluate the answers for the questions; if no source is mentioned, the answer is based on expert opinion.

^c^ Questions to assess economic, socio-ethical or environmental impact can also be answered with ‘absent’ if no impact is expected at all.

**References**

1. OIE (World Organisation for Animal Health). World Animal Health Information System. 2020 [Cited 2020 April 16]. Available from: <https://www.oie.int/wahis_2/public/wahid.php/Wahidhome/Home>
2. Zientara S, Weyer CT, Lecollinet S. African horse sickness. Rev Sci Tech Off Int Epiz. 2015; 34(2): 315-327.
3. Venter GJ, Koekemoer JJO, Paweska JT. Investigations on outbreaks of African horse sickness in the surveillance zone in South Africa. Rev Sci Tech Off Int Epiz. 2006; 25(3): 1097-1109.
4. Faverjon C, Leblond A, Hendrikx P, Balenghien T, De Vos CJ, Fischer EAJ, et al. A spatiotemporal model to assess the introduction risk of African horse sickness by import of animals and vectors in France. BMC Vet Res. 2015; 11: 127. doi: 10.1186/s12917-015-0435-4
5. De Vos CJ, Hoek CA, Nodelijk G. Risk of introducing African horse sickness virus into the Netherlands by international equine movements. Prev Vet Med. 2012; 106: 108-122. doi:10.1016/j.prevetmed.2012.01.019.
6. Eurostat. Comext Bulk Download; 2019 [Cited 2019 April 19]. Available from: <https://ec.europa.eu/eurostat/estat-navtree-portlet-prod/BulkDownloadListing?sort=1&dir=comext>
7. Möhlmann TWR, Bekendam AM, Van Kemenade I, Wennergren U, Favia G, Takken W, et al. Latitudinal diversity of biting midge species within the Obsoletus group across three habitats in Europe. Med Vet Entomol. 2019; 33: 420-426. doi: 10.1111/mve.12379.
8. Backer JA, Nodelijk G. Transmission and Control of African Horse Sickness in The Netherlands: A Model Analysis. PLoS ONE 2011; 6(8): e23066. doi: 10.1371/journal.pone.0023066.
9. Braks M, Mancini G, Swart M, Goffredo M. Risk of vector-borne diseases for the EU: Entomological aspects: Part 2. EFSA supporting publication 2017; 2017:EN-1184. doi: 10.2903/sp.efsa.2017.EN-1184.
10. Elbers ARW, Meiswinkel, R. Culicoides (Diptera: Ceratopogonidae) and livestock in the Netherlands: comparing host preference and attack rates on a Shetland pony, a dairy cow, and a sheep. J Vector Ecol. 2015; 40(2): 308-317. doi: 10.1111/jvec.12169.
11. Elbers, ARW, Gonzales, JL, Meiswinkel R. Comparing Culicoides biting rates in horses and cattle in The Netherlands: potential relevance to African horse sickness epidemiology. Entomol Exp Appl. 2018; 166: 535-544. doi: 10.1111/eea.12684.
12. KNHS (Koninklijke Nederlandse Hippische Sportfederatie). Kerncijfers paardensport. 2016 [Cited 2021 Feb 25]. Available from: <https://www.knhs.nl/kennisbank/publicaties/brochure-nederland-paardenland/> (in Dutch)
13. Mourits MCM, Saatkamp HW. Kostenberekening van een uitbraak met Afrikaanse paardenpest in Nederland. Wageningen, The Netherlands: Business Economics, Wageningen University; 2010. (in Dutch)
14. CBS. Statistics Netherlands, Statline, Agricultural Census; 2019 [Cited 2020 April 16]. Available from: <https://opendata.cbs.nl/statline/#/CBS/nl/>
15. EFSA AHAW Panel (EFSA Panel on Animal Health and Welfare), More S, Bicout D, Bøtner A, Butterworth A, Calistri P, et al. Scientific opinion on vector-borne diseases. EFSA Journal 2017; 15(5):4793. doi: 10.2903/j.efsa.2017.4793.
16. Sabirovic M, López M, Patel K, Kingston A, Hall S. African horse sickness: Potential risk factors and the likelihood for the introduction of the disease to the United Kingdom. London, United Kingdom: Defra. 2008 Nov 6 [Cited 2021 Feb 23]. Available from: <https://www.yumpu.com/en/document/read/16949542/african-horse-sickness-potential-risk-factors-and-archive-defra>
17. Koenraadt CJM, Balenghien T, Carpenter S, Ducheyne E, Elbers ARW, Fife M, et al. Bluetongue, Schmallenberg - what is next? Culicoides-borne viral diseases in the 21st Century. BMC Vet Res. 2014; 10: 77. doi: 10.1186/1746-6148-10-77.
18. EFSA AHAW Panel (EFSA Panel on Animal Health and Welfare). Scientific Opinion on Epizootic Hemorrhagic Disease. EFSA Journal 2009; 7(12): 1418. doi:10.2903/j.efsa.2009.1418.
19. De Vos C, Hoek M, Fischer E, De Koeijer A. Bremmer J. Risk assessment framework for emerging vector-borne livestock diseases. Report 11-CVI0168. Lelystad, the Netherlands: Central Veterinary Institute, part of Wageningen UR; 2011 [Cited 2018 Feb 15]. Available from: https://edepot.wur.nl/198115.
20. Savini G, Afonso A, Mellor P, Aradaib I, Yadin H, Sanaa M, et al. Epizootic haemorrhagic disease. Res Vet Sci. 2011; 91: 1-17. doi: 10.1016/j.rvsc.2011.05.004.
21. Barnard BJH, Gerdes GH, Meiswinkel R. Some epidemiological and economic aspects of a bluetongue-like disease in cattle in South Africa – 1995/96 and 1997. Onderstepoort J Vet Res. 1998; 65: 145–151.
22. Paweska JT, Venter, GJ, Hamblin C. A comparison of the susceptibility of *Culicoides imicola* and *C. bolitinos* to oral infection with eight serotypes of epizootic haemorrhagic disease virus. Med Vet Entomol. 2005; 19, 200-207. doi: 10.1111/j.0269-283X.2005.00560.x.
23. Santman-Berends IMGA, Stegeman JA, Vellema P, Van Schaik G. Estimation of the reproduction ratio (R0) of bluetongue based on serological field data and comparison with other BTV transmission models. Prev Vet Med. 2013; 108: 276-284. doi: 10.1016/j.prevetmed.2012.11.004.
24. Kedmi M, Levi S, Galon N, Bomborov V, Yadin H, Batten C, et al. No evidence for involvement of sheep in the epidemiology of cattle virulent epizootic hemorrhagic disease virus. Vet Microbiol. 2011; 148: 408-412. doi: 10.1016/j.vetmic.2010.09.015.
25. Ruder MG, Lysyk TJ, Stallknecht DE, Foil LD, Johnson DJ, Chase CC, et al. Transmission and Epidemiology of Bluetongue and Epizootic Hemorrhagic Disease in North America: Current Perspectives, Research Gaps, and Future Directions. Vector Borne Zoonotic Dis. 2015; 15(6): 348-363. doi: 10.1089/vbz.2014.1703.
26. Napp S, Gubbins S, Calistri P, Allepuz A, Alba A, García-Bocanegra I, et al. Quantitative assessment of the probability of bluetongue virus overwintering by horizontal transmission: application to Germany. Vet Res. 2011; 42: 4. doi: 10.1186/1297-9716-42-4.
27. Elbers ARW, Backx A, Mintiens K, Gerbier G, Staubach C, Hendrickx G, et al. Field observations during the Bluetongue serotype 8 epidemic in 2006. II. Morbidity and mortality rate, case fatality and clinical recovery in sheep and cattle in the Netherlands. Prev Vet Med. 2008; 87: 31-40. doi: 10.1016/j.prevetmed.2008.06.003.
28. Velthuis AGJ, Saatkamp HW, Mourits MCM, De Koeijer AA, Elbers ARW. Financial consequences of the Dutch bluetongue serotype 8 epidemics of 2006 and 2007. Prev Vet Med. 2010; 93: 294-304. doi: 10.1016/j.prevetmed.2009.11.007.
29. OIE (World Organisation for Animal Health). Epizootic haemorrhagic disease. Technical disease card. 2019 Dec [Cited 2020 July 30]. Available from: <https://www.oie.int/fileadmin/Home/eng/Animal_Health_in_the_World/docs/pdf/Disease_cards/EPIZOOTIC_HEAMORRHAGIC_DISEASE.pdf>
30. Nielsen SS, Alvarez J, Bicout DJ, Calistri P, Depner K, Drewe JA, et al. Rift Valley Fever – epidemiological update and risk of introduction into Europe. EFSA Journal 2020; 18(3): 6041. doi: 10.2903/j.efsa.2020.6041.
31. Pepin M, Bouloy M, Bird BH, Kemp A, Paweska J. Rift Valley fever virus (Bunyaviridae: Phlebovirus): an update on pathogenesis, molecular epidemiology, vectors, diagnostics and prevention. Vet Res. 2010; 41: 61. doi: 10.1051/vetres/2010033.
32. Jeanmaire EM, Rabenarivahiny R, Biarmann M, Rabibisoa L, Ravaomanana F, Randriamparany T, et al. Prevalence of Rift Valley Fever Infection in Ruminants in Madagascar After the 2008 Outbreak. Vector Borne Zoonotic Dis. 2011; 11(4): 395-402. doi: 10.1089/vbz.2009.0249.
33. Fafetine JM, Coetzee P, Mubemba B, Nhambirre O, Neves L, Coetzer JAW, et al. Rift Valley Fever Outbreak in Livestock, Mozambique, 2014. Emerg Infect Dis. 2016; 22(12): 2165-2167. doi: 10.3201/eid2212.160310.
34. Chevalier V, Thiongane Y, Lancelot R. Endemic Transmission of Rift Valley Fever in Senegal. Transbound Emerg Dis. 2009; 56: 372-374. doi: 10.1111/j.1865-1682.2009.01083.x.
35. CBS. Statistics Netherlands, Statline, Zeevaart; reis- en ladinggegevens per kwartaal, 1996 – 2010; 2012 Aug 17 [Cited 2020 May 27]. Available from: <https://opendata.cbs.nl/statline/#/CBS/nl/dataset/00374hvv/table?ts=1590565077220> (in Dutch)
36. Brown EBE, Adkin A, Fooks AR, Stephenson B, Medlock JM, Snary EL. Assessing the Risks of West Nile Virus–Infected Mosquitoes from Transatlantic Aircraft: Implications for Disease Emergence in the United Kingdom. Vector Borne Zoonotic Dis. 2012; 12(4): 310-320. doi: 10.1089/vbz.2010.0176.
37. Scholte EJ, Ibáñez-Justicia A, Stroo A, de Zeeuw J, Den Hartog W, Reusken CBEM. Mosquito collections on incoming intercontinental flights at Schiphol International airport, the Netherlands, 2010-2011. J Eur Mosq Control Assoc. 2014; 32: 17-21.
38. Oliveira ARS, Piaggio J, Cohnstaedt LW, McVey DS, Cernicchiaro N. A quantitative risk assessment (QRA) of the risk of introduction of the Japanese encephalitis virus (JEV) in the United States via infected mosquitoes transported in aircraft and cargo ships. Prev Vet Med. 2018; 160: 1-9. doi: 10.1016/j.prevetmed.2018.09.020.
39. Van Bortel W, Petric D, Ibáñez Justicia A, Wint W, Krit M, Mariën J, et al. Assessment of the probability of entry of Rift Valley fever virus into the EU through active or passive movement of vectors. EFSA Supporting Publication 2020; 2020:EN-1801. doi: 10.2903/sp.efsa.2020.EN-1801.
40. Linthicum KJ, Davies FG, Kairo A, Bailey CL. Rift Valley Fever Virus (Family Bunyaviridae, Genus *Phlebovirus*). Isolations from Diptera Collected during an Inter-Epizootic Period in Kenya. J Hyg Camb. 1985; 95(1): 197-209. doi: 10.1017/s002217240006243.
41. Fischer EAJ, Boender GJ, Nodelijk G, De Koeijer AA, Van Roermund HJW. The transmission potential of Rift Valley fever virus among livestock in the Netherlands: a modelling study. Vet Res. 2013; 44: 58. doi: 10.1186/1297-9716-44-58.
42. Dórea FC, Swanenburg M, Van Roermund H, Horigan V, De Vos C, Gale P, et al. Data collection for risk assessments on animal health. EFSA supporting publication 2017; 2017:EN-1171. doi: 10.2903/sp.efsa.2017.EN-1171.
43. Ibañez-Justicia A, Stroo A, Dik M, Beeuwkes J, Scholte EJ. National Mosquito (Diptera: Culicidae) Survey in The Netherlands 2010–2013. J Med Entomol. 2015; 52(2): 185-198. doi: 10.1093/jme/tju058.
44. Hoek MR, Fischer EAJ, De Koeijer AA, Bremmer J, De Vos CJ. Risk assessment framework for exotic vector-borne livestock diseases; a Rift Valley fever case study. Report number 11-CVI0412. Lelystad, The Netherlands: Central Veterinary Institute, Wageningen UR; 2011.
45. Antonis AFG, Kortekaas J, Kant J, Vloet RPM, Vogel-Brink A, Stockhofe N, et al. Vertical Transmission of Rift Valley Fever Virus Without Detectable Maternal Viremia. Vector Borne Zoonotic Dis. 2013; 13(8): 601-606. doi: 10.1089/vbz.2012.1160.
46. McMillen CM, Hartman AL. Rift Valley fever in animals and humans: Current perspectives. Antiviral Res. 2018; 156: 29-37. doi: 10.1016/j.antiviral.2018.05.009.
47. Barzon L, Pacenti M, Frachin E, Squarzon L, Lavezzo E, Cattai M, et al. The Complex Epidemiological Scenario of West Nile Virus in Italy. Int J Environ Res Public Health 2013; 10: 4669-4689. doi: 10.3390/ijerph10104669.
48. Roberts H, Crabb J. West Nile virus: Potential risk factors and the likelihood for introduction into the United Kingdom. 2012 May 1 [Cited 2021 Feb 23]. Available from: <http://webarchive.nationalarchives.gov.uk/20140507133914/http://www.defra.gov.uk/animal-diseases/files/qra-wnv-120501.pdf>
49. Koenraadt CJM, Möhlmann TWR, Verhulst NO, Spitzen J, Vogels CBF. Efect of overwintering on survival and vector competence of the West Nile virus vector Culex pipiens. Parasit Vectors 2019; 12: 147. doi: 10.1186/s13071-019-3400-4.
50. Chitnis N, Hyman JM, Manore CA. Modelling vertical transmission in vector-borne diseases with applications to Rift Valley fever 2013. J Biol Dyn. 2013; 7(1): 11-40. doi: 10.1080/17513758.2012.733427.
51. Peyre M, Chevalier V, Abdo-Salem S, Velthuis A, Antoine-Moussiaux N, Thiry E, et al. A Systematic Scoping Study of the Socio-Economic Impact of Rift Valley Fever: Research Gaps and Needs. Zoonoses Public Health 2015; 62: 309-325. doi: 10.1111/zph.12153.
52. OIE (World Organisation for Animal Health). Rift Valley fever. Technical disease card. 2019 Dec [Cited 2021 Feb 25]. Available from: <https://www.oie.int/fileadmin/Home/eng/Animal_Health_in_the_World/docs/pdf/Disease_cards/RIFT_VALLEY_FEVER.pdf>
53. Huirne RBM, Mourits M, Tomassen F, De Vlieger JJ, Vogelzang TA. MKZ: Verleden, Heden en Toekomst. Over de preventie en bestrijding van MKZ. Rapport 6.02.14, Den Haag, The Netherlands: LEI; 2001. (in Dutch)
54. Sambri V, Capobianchi M, Charrel R, Fyodorova M, Gaibani P, Gould E, et al. West Nile virus in Europe: Emergence, epidemiology, diagnosis, treatment, and prevention. Clin Microbiol Infect. 2013; 19: 699-704. doi: 10.1111/1469-0691.12211.
55. Durand B, Balança G, Baldet T, Chevalier V. A metapopulation model to simulate West Nile virus circulation in Western Africa, Southern Europe and the Mediterranean basin. Vet Res. 2010; 41: 32. doi: 10.1051/vetres/2010004.
56. Durand B, Tran A, Balança G, Chevalier V. Geographic variations of the bird-borne structural risk of West Nile virus circulation in Europe. PLoS ONE 2017; 12(10): e0185962. doi: 10.1371/journal.pone.0185962.
57. Bessell PR, Robinson RA, Golding N, Searle KR, Handel IG, Boden LA, et al. Quantifying the Risk of Introduction of West Nile Virus into Great Britain by Migrating Passerine Birds. Transbound Emerg Dis. 2014; 63(5): e347-e359. doi: 10.1111/tbed.12310.
58. Martínez-de la Puente J, Ferraguti M, Ruiz S, Roiz D, Llorente F, Pérez-Ramírez E, et al. Mosquito community influences West Nile virus seroprevalence in wild birds: implications for the risk of spillover into human populations. Sci Rep. 2018; 8: 2599. doi: 10.1038/s41598-018-20825-z.
59. Eybpoosh S, Fazlalipour M, Baniasadi V, Pouriayevali MH, Sadeghi F, Vasmehjani AA, et al. Epidemiology of West Nile Virus in the Eastern Mediterranean region: A systematic review. PLoS Negl Trop Dis. 2019; 13(1): e0007081. doi: 10.1371/journal.pntd.0007081.
60. McMillan JR, Blakney RA, Mead DG, Koval WT, Coker SM, Waller LA, et al. Linking the vectorial capacity of multiple vectors to observed patterns of West Nile virus transmission. J Appl Ecol. 2019; 56: 956-965. doi: 10.1111/1365-2664.13322.
61. Knap N, Korva M, Ivović V, Kalan K, Jelovšek M, Sagadin M, et al. West Nile Virus in Slovenia. Viruses 2020; 12:720. doi:10.3390/v12070720.
62. Angelini P, Tamba M, Finarelli AC, Bellini R, Albieri A, Bonilauri P, et al. West Nile virus circulation in Emilia-Romagna, Italy: the integrated surveillance system 2009. Euro Surveill. 2010; 15(16): 19547. doi: 10.2807/ese.15.16.19547.
63. Calzolari M, Bonilauri P, Bellini R, Albieri A, Defilippo F, Maioli G, et al. Evidence of Simultaneous Circulation of West Nile and Usutu Viruses in Mosquitoes Sampled in Emilia-Romagna Region (Italy) in 2009. PLoS ONE 2010; 5(12): e14324. doi:10.1371/journal.pone.0014324.
64. Engler O, Savini G, Papa A, Figuerola J, Groschup MH, Kampen H, et al. European Surveillance for West Nile Virus in Mosquito Populations. Int J Environ Res Public Health 2013; 10: 4869-4895. doi: 10.3390/ijerph10104869.
65. Pradier S, Lecollinet S, Leblond A. West Nile virus epidemiology and factors triggering change in its distribution in Europe. Rev Sci Tech Off Int Epiz. 2012; 31(3): 829-844.
66. Cruz-Pacheco G, Esteva L, Montaño-Hirose JA, Vargas C. Modelling the dynamics of West Nile Virus. Bull Math Biol. 2005; 67: 1157-1172. doi: 10.1016/j.bulm.2004.11.008.
67. Wonham MJ, Lewis MA, Renclawowicz J, Van den Driessche P. Transmission assumptions generate conflicting predictions in host–vector disease models: a case study in West Nile virus. Ecol Lett. 2006; 9: 706-725. doi: 10.1111/j.1461-0248.2006.00912.x.
68. Hartemink NA, Davis SA, Reiter P, Hubálek Z, Heesterbeek JAP. Importance of Bird-to-Bird Transmission for the Establishment of West Nile Virus. Vector Borne Zoonotic Dis. 2007; 7(4): 575-584. doi: 10.1089/vbz.2006.0613.
69. Pawelek KA, Niehaus P, Salmeron C, Hager EJ, Hunt GJ. Modeling Dynamics of *Culex pipiens* Complex Populations and Assessing Abatement Strategies for West Nile Virus. PLoS ONE 2016; 9(9): e108452. doi:10.1371/journal.pone.0108452,
70. Vogels CBF, Hartemink N, Koenraadt CJM. Modelling West Nile virus transmission risk in Europe: effect of temperature and mosquito biotypes on the basic reproduction number. Sci Rep. 2017; 7: 5022. doi: 10.1038/s41598-017-05185-4.
71. Kioutsioukis I, Stilianakis NI. Assessment of West nile virus transmission risk from a weather-dependent epidemiological model and a global sensitivity analysis framework. Acta Trop. 2019; 193: 129-141. doi: 10.1016/j.actatropica.2019.03.003.
72. Komar N, Langevin S, Hinten S, Nemeth N, Edwards E, Hettler D, et al. Experimental Infection of North American Birds with the New York 1999 Strain of West Nile Virus. Emerg Infect Dis. 2003; 9(3): 311-322. doi: 10.3201/eid0903.020628.
73. Sovon. Bird Atlas of the Netherlands. 2018 Nov 24 [Cited 2020 July 30]. Available from: <https://www.vogelatlas.nl/>
74. Montecino-Latorre D, Barker CM. Overwintering of West Nile virus in a bird community with a communal crow roost. Sci Rep. 2018; 8: 6088. doi: 10.1038/s41598-018-24133-4.
75. Rudolf I, Betášová L, Blažejová H, Venclíková K, Straková P, Šebesta O, et al. West Nile virus in overwintering mosquitoes, central Europe. Parasit Vectors 2017; 10:452. doi: 10.1186/s13071-017-2399-7.
76. Nasci RS, Savage HM, White DJ, Miller JR, Cropp BC, Godsey MS, et al. West Nile virus in overwintering Culex mosquitoes, New York City, 2000. Emerg Infect Dis. 2001; 7: 742–744. doi: 10.3201/eid0704.017426.
77. Baqar S, Hayes CG, Murphy JR, Watts DM. Vertical transmission of West Nile virus by *Culex* and *Aedes* species mosquitoes. Am J Trop Med Hyg. 1993; 48(6): 757-762. doi: 10.4269/ajtmh.1993.48.757.
78. Dohm DJ, Sardelis MR, Turell MJ. Experimental Vertical Transmission of West Nile Virus by *Culex pipiens* (Diptera: Culicidae). J Med Entomol. 2002; 39(4): 640-644. doi: 10.1603/0022-2585-39.4.640.
79. Goddard LB, Roth AE, Reisen WK, Scott TW. Vertical Transmission of West Nile Virus by Three California *Culex* (Diptera: Culicidae) Species. J Med Entomol. 2003; 40(6): 743-746. doi: 10.1603/0022-2585-40.6.743.
80. Fortuna C, Remoli ME, Di Luca M, Severini F, Toma L, Benedetti E, et al. Experimental studies on comparison of the vector competence of four Italian *Culex pipiens* populations for West Nile virus. Parasit Vectors 2015; 8: 463. doi: 10.1186/s13071-015-1067-z.
81. ADNS (Animal Disease Notification System), 2019. Overview of animal disease info from 2018. Report summary. 2019 Jan 7 [Cited 2020 Jan 13]. Available from: https://ec.europa.eu/food/sites/food/files/animals/docs/ad_adns_overview_2018.pdf
82. ECDC (European Centre for Disease Prevention and Control). Epidemiological update: West Nile virus transmission season in Europe, 2018. 2018 Dec 14 [Cited 2020 Jan 13] Available from: https://www.ecdc.europa.eu/en/news-events/epidemiological-update-west-nile-virus-transmission-season-europe-2018
